# Cessation of Pulsed Lavage During the SARS-CoV-2 Pandemic: The Effect on Hip Hemiarthroplasty Cement Mantles

**DOI:** 10.7759/cureus.16809

**Published:** 2021-08-01

**Authors:** Dhiraj Sharma, Kate Spacey, Vivek Sharma, Alastair Vince

**Affiliations:** 1 Department of Trauma and Orthopaedics, Addenbrooke’s Hospital, Cambridge University Hospitals NHS Foundation Trust, Cambridge, GBR; 2 Department of Trauma and Orthopaedics, Norfolk and Norwich University Hospitals Foundation Trust, Norwich, GBR

**Keywords:** hip hemiarthroplasty, neck of femur fracture, covid-19, sars-cov-2

## Abstract

Background

With the severe acute respiratory syndrome coronavirus 2 (SARS-CoV-2) pandemic, we were issued with guidance to minimize aerosol-generating procedures and discontinued the use of pulsed lavage for hip hemiarthroplasty. Instead, we used a bladder syringe to wash the femoral canal. The aim of this study was to assess whether this change in practice had a detrimental effect on the quality of the bone cement mantles in patients undergoing cemented hip hemiarthroplasty.

Methodology

We performed a retrospective review of all patients treated at a tertiary teaching hospital in the United Kingdom (Addenbrookes, Cambridge University Hospitals) presenting with a neck of femur fracture requiring a hemiarthroplasty between October 2019 and June 2020. We retrospectively assessed 100 post-operative radiographs for patients who had received hip hemiarthroplasty following neck of femur fragility fracture (50 before the service change and 50 after). The Barrack classification was used to assess the quality of the bone cement mantle.

Results

Pre-SARS-CoV-2, 30% of hemiarthroplasties were deemed as being “at risk” of aseptic loosening. During SARS-CoV-2, 64% of hips were deemed as being “at risk.” This represents a statistically significant absolute increase of 34% (P < 0.05, the P value is 0.000645). Both clinicians agreed on the classification of hips “at risk” or “not at risk” (i.e., grades C/D and A/B, respectively) in 85% of the cases. Cohen’s kappa coefficient was calculated as 0.68, indicating substantial agreement.

Conclusions

Following our experience of this forced service change, we would discourage abandoning the use of pulsed lavage in future pandemics. We have demonstrated an association between abandoning pulsed lavage and detrimental effects on the procedural quality for hip hemiarthroplasty. Patients treated over this time period will be closely monitored for operative complications. As this was the only equipment change made for this procedure, we have demonstrated its detrimental effect on the procedural quality. Should pulsed lavage be discontinued, patients may need to be counseled for higher risk of early failure and revision surgery and may require long-term radiographic follow-up. In SARS-CoV-2-positive patients, Surgeons should carefully consider the risks and benefits of using pulsed lavage in accordance with the personal protective equipment they have available and the consequential impact on the bone cement mantle quality.

## Introduction

The severe acute respiratory syndrome coronavirus 2 (SARS-CoV-2) pandemic posed an unknown risk to the world and led to a wave of unprecedented changes in healthcare delivery. Many of these changes were made in operating theater practice, an environment where aerosol-generating procedures (AGPs) are commonly performed.

In attempts to reduce AGPs during the SARS-CoV-2 pandemic, the use of pulsed lavage was discontinued in our hospital and replaced by lavage with a bladder syringe and saline [1]. From clinical observation, this did not adequately cleanse the canal of blood or fat. For this procedure, this was the only equipment change made.

Femoral preparation and cementing techniques have seen generational improvements and we currently utilize fourth-generation techniques in the elective setting; however, a modified version of cementing is utilized in fragile hip fracture patients to reduce the risk of bone cement implantation syndrome (BCIS) [2]. Pulsed lavage is used to clean the femoral canal prior to retrograde cement insertion; an evidence-based strategy for improving cement mantle quality and reducing implant-associated infections [3,4]. Thorough femoral preparation using pulsed lavage is recommended by the National Patient Safety Agency 2009 guidelines as a means of reducing the incidence of BCIS [5]. Preparation of the femoral canal requires adequate removal of debris, fat, and blood from cancellous bone to allow for the bone cement to interdigitate. This improves the cement mantle and reduces the risk of fat being forcibly displaced into the capillary system.

Due to the generalized frailty and comorbidities associated with the fragility fracture population, pressurization of the cement mantle is not routinely performed. Although it improves the quality of the bone cement mantle, it increases the risk of BCIS in this population [6]. This element of the cementing technique remained unchanged during the SARS-CoV-2 pandemic, as such we would not expect complete “white-out,” perfect cement mantles to be seen on post-operative radiographs of those undergoing cemented hemiarthroplasty for hip fractures.

The aim of this study was to assess the impact of discontinuing pulsed lavage on the quality of the bone cement mantle in patients undergoing hip hemiarthroplasty during the SARS-CoV-2 pandemic.

This article was previously presented as a poster at the 2021 Association of Surgeons In Training annual conference on March 5-7, 2021.

## Materials and methods

We performed a retrospective review of all patients treated at a tertiary teaching hospital in the United Kingdom (Addenbrookes, Cambridge University Hospitals). The study population was defined as patients who had sustained a neck of femur fracture requiring a hemiarthroplasty between October 2019 and June 2020. A post-hoc power calculation, with confidence intervals of 80% and a type 1 error rate of 5%, determined that during each time period, a sample size of 28 patients would be appropriate in each group. In total, 50 consecutive radiographs for hip hemiarthroplasties were reviewed before and during the SARS-CoV-2 service change (100 in total). All patients were over 60 years old and underwent hemiarthroplasties utilizing Stryker Exeter stem prothesis. Exclusion criteria was any non-fragility fracture and patients under 60 years old. Patients were determined as requiring hip hemiarthroplasty following a multidisciplinary review of the patients and their records in a trauma meeting. All operations were performed by, or under the supervision, of a senior orthopedic trainee (registrar) or consultant. Full personal protective equipment was used by the operating surgeon throughout all procedures.

Patients were identified using the hospital’s electronic theater management system (Epic) to identify those undergoing cemented hemiarthroplasty during the referenced time periods.

These records were manually checked against patient operating lists. Thereafter, post-operative clinical information was gathered from a retrospective review of Picture Archiving and Communication Systems. Post-operative radiographs of hemiarthroplasties performed before and during the SARS-CoV-2 pandemic, under the supervision of multiple consultant surgeons, were independently reviewed by a senior orthopedic registrar or consultant. The Barrack classification was used to grade cement quality as A-D (Table 1).

**Table 1:** Barrack classification for cemented hip prosthesis.

Barrack grading	Radiographic characteristics
A	“White-out” – No radiolucent lines between the bone and cement
B	Radiolucent lines seen in up to 50% of the bone cement mantle
C	Radiolucent lines seen in greater than 50% of the bone cement mantle
D	Radiolucent lines seen around the entire bone cement mantle and/or absence of cement distal to the stem

“At risk” hips were defined as Barrack classification C and D as they were at a higher risk of aseptic prosthesis loosening. The following demographic information was also recorded: patients’ age at the time of operation, date of operation, American Society of Anesthesiologists (ASA) grade, and primary surgeon grades.

Data analysis was accomplished using a spreadsheet on Microsoft Excel 2020. Categorical data, such as Barrack classification, were summarized as numbers (proportions), and comparisons were made using the Yates’ Chi-squared test. As the study was purely retrospective, and no patients were individually identifiable, NHS research ethics committee approval was not required. However, the study was registered with the hospital’s audit department.

## Results

A total of 100 patients were included in the study. Radiographs of 50 patients undergoing hip hemiarthroplasty were reviewed from the pre-SARS-CoV-2 period. The average age was 84.9 years (range: 67-97 years) and average ASA was 3 (range: 2-4). Consultants, registrars, and senior house officers were the documented primary surgeon in 16%, 74%, and 10% of the cases, respectively. “At risk” hips (Grades C-D) were identified in 30% of the cases (n = 15).

Radiographs of 50 patients undergoing hemiarthroplasties for hip fractures, were reviewed and graded during the period that pulsed lavage was halted. The average age was 83.3 years (range: 61-97 years) and average ASA was 3 (range: 2-4). Consultants, registrars, and senior house officers were the documented primary surgeon in 14%, 82%, and 4% of the cases, respectively. “At risk” hips were identified in 64% of the radiographs (n = 32). Patient ASA grades are displayed in Table 2.

**Table 2:** Patient ASA grades and proportion with procedures producing hip hemiarthroplasties “at risk” of aseptic loosening. SARS-CoV-2, severe acute respiratory syndrome coronavirus 2; ASA, American Society of Anesthesiologists

	Pre-SARS-CoV-2		SARS-CoV-2		
ASA grade	Total	Proportion “at risk” (%)	Total		Proportion “at risk” (%)
2	12	5 (41.7%)	12		8 (66.7%)
3	28	7 (38.9%)	34		23 (67.6%)
4	10	3 (30%)	4		1 (25%)

Procedure primary surgeons were classified by seniority (junior to senior) as senior house officer, registrar, or consultant (Table 3).

**Table 3:** Primary surgeon title and associated procedures producing hip hemiarthroplasties deemed as being “at risk” of aseptic loosening. SARS-CoV-2, severe acute respiratory syndrome coronavirus 2

	Pre-SARS-CoV-2		SARS-CoV-2	
Primary surgeon	Total procedures	Procedures “at risk” (%)	Total procedures	Procedures “at risk”
Senior house officer	5	1 (20%)	2	0 (0%)
Registrar	37	9 (24.3%)	41	26 (63.4%)
Consultant	8	5 (62.5%)	7	4 (57%)

There was a statistically significant increase (34% absolute, 113.33% relative) in “at risk” hips performed during the service change (p = 0.000645). Using Yates’ Chi-squared calculation for a P < 0.05, the P-value was 0.000645. The two senior surgeons grading the hips agreed on the classification of hips “at risk” or “not at risk” (i.e., grades C/D and A/B, respectively) in 85% of the cases. Cohen’s kappa coefficient was calculated as 0.68, indicating substantial agreement.

Of those patients included during the pandemic, 90% underwent SARS-CoV-2 testing and no positive results were reported. There was no documented evidence of BCIS within the anesthetic records in either time period.

## Discussion

The SARS-CoV-2 pandemic has had an unprecedented impact on surgery affecting staffing, patient lists, patient care, peri-operative practice, and surgical training [7]. Nosocomial spread is a significant cause of hospital transmission, hence, the implementation of strict precautions surrounding AGPs [8,9]. Close evaluation of service changes has been imperative to minimize the detrimental sequelae.

Hip fractures are the most common cause for an orthopedic admission in the United Kingdom; hence, any increase in complication rates is likely to have a significant impact on patient care and service provision [10]. Pulsed lavage for femoral preparation improves the quality of the bone cement mantle and reduces the risk of infection and aseptic loosening, the most common causes for revision surgery [11]. The quality of the bone cement mantle can be assessed radiographically by evaluating the Gruen zones surrounding the prosthesis [12-15]. The Barrack classification assesses these zones for lucency and, as such, poor Barrack scores (C and D) are associated with aseptic loosening and early prosthesis failure [16,17].

Patient demographics between each group were similar, both by age and ASA grade. Prevalence of registrar and consultant lead procedures were similar across both groups. “At risk” (Barrack C and D) hips were identified in the pre-SARS-CoV-2 period; however, this was anticipated because we do not routinely pressurize the femoral canal at the time of cementation in frail hip fracture patients to reduce the risk of BCIS.

Our results demonstrate a statistically significant increase in the number of hemiarthroplasties graded as Barrack C and D (“at risk” of aseptic loosening) when we stopped using pulsed lavage. The grading clinicians were in agreement classifying “at risk” (Barrack C and D) or “not at risk” (Barrack A and B) hips in 85% of the cases, achieving a Cohen’s kappa coefficient that suggests substantial agreement. The increase in “at risk” hips was predominantly seen in registrar-performed procedures, increasing from 24.3% to 63.4% of the procedures. The registrars performed the majority of procedures in both time periods; therefore, any complication as a result of this equipment change is most likely to be reflected in their operations. We did not record the exact registrar grade and therefore cannot make inferences about experience and procedural performance; we accept this as a limitation of this study.

The majority of “at risk” hips were identified in patients with lower ASA grades (2 or 3). If these patients were to develop symptoms of failure, they would likely be fit enough and suitable for further surgical intervention to revise the initial hemiarthroplasty to a total hip replacement. With further changes in orthopedics and general practice, referral processes in response to the pandemic, and delays in resuming elective work, these post-operative patients may experience delays in receiving orthopedic assessment if they were to develop symptoms from early failure in the first year post-operatively.

Although pulsed lavage was deemed as being an AGP, there is currently no clear evidence demonstrating this. It would be beneficial for researchers to assess which common surgical procedures are associated with AGPs so evidence-based guidelines can be developed.

## Conclusions

Following our experience of this forced service change, we would discourage abandoning the use of pulsed lavage in future pandemics. We have demonstrated an association between pulsed lavage and the procedural quality for hip hemiarthroplasties in our population. Patients treated over this time period will be closely monitored for post-operative complications. Should pulsed lavage be discontinued, the patient may need to be counseled for higher risk of early failure and revision surgery and may require long-term radiographic follow-up. In SARS-CoV-2-positive patients, surgeons should carefully consider the risks and benefits of using pulsed lavage in accordance with the personal protective equipment they have available and the consequential impact on the bone cement mantle quality.
